# Should exercises be painful in the management of chronic musculoskeletal pain? A systematic review and meta-analysis

**DOI:** 10.1136/bjsports-2016-097383

**Published:** 2017-06-08

**Authors:** Benjamin E Smith, Paul Hendrick, Toby O Smith, Marcus Bateman, Fiona Moffatt, Michael S Rathleff, James Selfe, Pip Logan

**Affiliations:** 1 Department of Physiotherapy, Derby Teaching Hospitals NHS Foundation Trust, Derby, UK; 2 Division of Rehabilitation and Ageing, School of Medicine, University of Nottingham, Nottingham, UK; 3 Division of Physiotherapy and Rehabilitation Sciences, School of Health Sciences, University of Nottingham, Nottingham University Hospitals (City Campus), Nottingham, UK; 4 University of East Anglia, Norwich, UK; 5 Research Unit for General Practice in Aalborg, Department of Clinical Medicine at Aalborg University, Aalborg, Denmark; 6 Department of Occupational Therapy and Physiotherapy, Department of Clinical Medicine, Aalborg University Hospital, Aalborg, Denmark; 7 Manchester Metropolitan University, Manchester, UK

**Keywords:** Systematic review, Meta-analysis, musculoskeletal pain, musculoskeletal disorder, treatment, exercise, effectiveness

## Abstract

**Background:**

Chronic musculoskeletal disorders are a prevalent and costly global health issue. A new form of exercise therapy focused on loading and resistance programmes that temporarily aggravates a patient’s pain has been proposed. The object of this review was to compare the effect of exercises where pain is allowed/encouraged compared with non-painful exercises on pain, function or disability in patients with chronic musculoskeletal pain within randomised controlled trials.

**Methods:**

Two authors independently selected studies and appraised risk of bias. Methodological quality was evaluated using the Cochrane risk of bias tool, and the Grading of Recommendations Assessment system was used to evaluate the quality of evidence.

**Results:**

The literature search identified 9081 potentially eligible studies. Nine papers (from seven trials) with 385 participants met the inclusion criteria. There was short- term significant difference in pain, with moderate quality evidence for a small effect size of −0.27 (−0.54 to −0.05) in favour of painful exercises. For pain in the medium and long term, and function and disability in the short, medium and long term, there was no significant difference.

**Conclusion:**

Protocols using painful exercises offer a small but significant benefit over pain-free exercises in the short term, with moderate quality of evidence. In the medium and long term there is no clear superiority of one treatment over another. Pain during therapeutic exercise for chronic musculoskeletal pain need not be a barrier to successful outcomes. Further research is warranted to fully evaluate the effectiveness of loading and resistance programmes into pain for chronic musculoskeletal disorders.

**PROSPERO registration:**

CRD42016038882.

## Background

Musculoskeletal disorders are one of the most prevalent and costly disorders globally.^1 2^ Low back pain is considered the leading cause of years lived with disability worldwide, ahead of conditions such as depression, diabetes, cardiovascular disease and cancer, with a global point prevalence of 9.4%.^3 4^ Neck pain and other musculoskeletal pain ranks fourth and sixth in terms of years lived with disability, with a global point prevalence of 5% and 8%, respectively.^5 6^ In the UK, an estimated one in four people suffer from chronic musculoskeletal disorders,^7^ with an estimated economic consequence of 8.8 million working days lost.^8^

Previous systematic reviews have assessed the effectiveness of various interventions for musculoskeletal disorders, including pharmaceutical therapies,^9–12^ psychological-based therapies^13–16^ and physical-based therapies, including manual therapy^17–19^ and exercise.^16 20–24^ These have all presented poor to moderate results in terms of effectiveness at improving pain and function, and have identified limitations in the quality of included trials when drawing conclusions.

There is a high level of uncertainty and lack of sufficient level 1 evidence on which to base treatment for people with musculoskeletal disorders. A systematic review of self-management interventions for chronic musculoskeletal pain concluded that strong evidence existed that changes in the psychological factors, self-efficacy and depression were predictors of outcomes, irrespective of the intervention delivered, and strong evidence existed that positive changes in patients’ pain catastrophising and physical activity were mediating factors.^25^ Experimental studies have also demonstrated that stimulus context and the emotional response to pain affect the experience of pain,^26–28^ and have led to the development of desensitisation interventions for chronic musculoskeletal disorders.^29–31^

It has been proposed that modern treatment therapies for chronic musculoskeletal pain and disorders should be designed around loading and resistance programmes targeting movements and activities that can temporarily reproduce and aggravate patients’ pain and symptoms.^31–33^ Pain does not correlate with tissue damage,^34^ and psychological factors such as catastrophising and fear avoidance behaviours play an important role in the shaping of the physiological responses to pain, and therefore the development and maintenance of chronic pain.^35^ It is thought that such an exercise programme could facilitate the reconceptualisation of pain by addressing fear avoidance and catastrophising beliefs within a framework of ‘hurt not equalling harm’.^36 37^ Through this, proponents support the prescription of exercises into pain for chronic musculoskeletal pain and disorders.^31 37 38^ We define ‘exercise into pain’ as a therapeutic exercise where pain is encouraged or allowed.

No previous systematic reviews have evaluated the effectiveness of exercises into pain for chronic musculoskeletal pain. Therefore the object of this review was to compare the effect of exercises into pain compared with non-painful exercises on pain, function or disability in patients with chronic musculoskeletal pain within randomised controlled trials (RCTs), specifically exercises that were prescribed with instructions for patients to experience pain, or where patients were told it was acceptable and safe to experience pain, and to compare any difference in contextual factors and prescription parameters of the prescribed exercise intervention.

## Methods

This systematic review followed the recommendations of the PRISMA statement,^39^ and was registered with the International Prospective Register of Systematic Reviews (PROSPERO; http://www.crd.york.ac.uk/prospero/, reference CRD42016038882).

### Search strategy

An electronic database search was conducted on titles and abstract from inception to October 2016 on the following databases: the Allied and Complimentary Medicine Database, the Cumulative Index to Nursing and Allied Health Literature, the Cochrane Library, Embase, Medline, SPORTDiscus and Web of Science. For the keywords and keywords search strategy used, please see table 1. The database searches were accompanied by hand searches of the reference list of included articles, and the grey literature and ongoing trials were searched using the following databases: Open Grey, WHO International Clinical Trials Registry Platform, ClinicalTrials.gov and the bjsports-2016-097383 portfolio.

**Table 1:** Search strategy

1	Randomised controlled trials as
2	Topic/
3	randomised controlled trial.pt
4	controlled clinical trial.pt
5	or/1-3
6	Exp Pain
7	Exp Musculoskeletal Disease
8	Exp Musculoskeletal Pain
9	Or/5-7
10	Rehabilitation
11	Bone
12	Joint
13	Muscle
14	Exp Exercise therapy
15	Physiotherapy
16	Physical therapy
17	Physical-therapy
18	Exp Exercise Or/9-17
19	(exercise adj7 pain$).af
20	High load
21	Loaded$
22	Resistance$
23	Eccentric$
24	Concentric$
25	Weight loaded
26	Weight-loaded
27	Weight resistance
28	Weight-resistance
29	High-load
30	Heavy load
31	Heavy-load
32	Direction$ preference
33	Directional-preference
34	Or/19-33
35	4 and 8 and 18 and 34 (limited to English)

For inclusion, the studies had to meet the following criteria: adults recruited from the general population with any musculoskeletal pain or disorder greater than 3 months; participants with pain suggestive of non-musculoskeletal pain, for example, headache, migraine, bowel/stomach pain, cancer, fibromyalgia, chest pain, and breathing difficulties were excluded. Studies had to have a primary treatment arm of therapeutic exercises that was advised to be purposively painful, or where pain was allowed or tolerated. The comparison group had to use therapeutic exercises that were pain-free. Included studies were required to report pain, disability or function. Studies had to be full RCT published in English. Studies that were not randomised or quasi-random were excluded.

### Study selection

One reviewer (BES) undertook the searches. Titles and abstracts were screened by one reviewer (BES), with potential eligible papers retrieved and independently screened by two reviewers (BES and PH). Initial inclusion agreement was 81%, and using Cohen’s statistic method the kappa agreement was *k*=0.47, which is considered ‘fair to moderate’ agreement.^40–42^ All initial disagreements were due to intervention criteria, specifically the levels of pain during the therapeutic exercises in each intervention arm,^43–50^ and were resolved through consensus. Three trials needed further information with regard to their control exercise to ascertain if they met the inclusion criteria, and all three were contacted.^51–53^ All three responded with further information, and after discussion there was consensus to include two of the three trials.^51 52^

### Data extraction

The following data were extracted from the included articles: trial design, participant information, intervention and control exercise, setting, follow-up periods and outcome data.^54^ The data were independently extracted and transcribed to a standard table by one reviewer (BES), and then 25% of the data were independently checked by a second reviewer (PH). Effectiveness was judged in the short term (≤3 months from randomisation), medium term (>3 and<12 months) and long term (≥12 months), as recommended by the 2009 Updated Method Guidelines for Systematic Reviews in the Cochrane Back Review Group.^55^

### Quality assessment

Each included study was appraised independently by two reviewers (BES and PH) for methodological quality using the Cochrane risk of bias tool for randomised clinical trials.^56^ The tool was originally developed in 2008, and updated in 2011, and is based on seven key bias domains^57^: sequence generation and allocation concealment (both within the domain of selection bias or allocation bias), blinding of participants and personnel (performance bias), blinding of outcome assessors (detection bias), incomplete outcome data (attrition bias) and selective reporting (reporting bias).^56^ For each domain the reviewers judged the risk of bias as ‘high’, ‘low’ or ‘unclear’. Percentage agreement between the two reviewers for the individual risk of bias domains for the Cochrane risk of bias tool was 86%, with a kappa of κ=0.76, which is considered ‘substantial or good’,^40–42^ and disagreements were resolved through consensus.

We used the Grading of Recommendations Assessment, Development and Evaluation (GRADE) system to rate the overall quality of the body of evidence in each pooled analysis.^58^ We did not evaluate the publication bias domain in this review as it is not recommended to assess funnel plot asymmetry with a meta-analysis of fewer than 10 trials.^59^ A GRADE profile was completed for each pooled estimate. Where only single trials were available, evidence from studies with <400 participants was downgraded for inconsistency and imprecision and rated as low-quality evidence. Three reviewers assessed these factors for each outcome and agreed by consensus (BES, PH and TOS).

The quality of evidence was defined as the following: (1) high quality—further research is unlikely to change our confidence in the estimate of effect; the Cochrane risk of bias tool identified no risks of bias and all domains in the GRADE classification were fulfilled; (2) moderate quality—further research is likely to have an important impact on our confidence in the estimate of effect, and one of the domains in the GRADE classification was not fulfilled; (3) low quality—further research is likely to have an important impact on our confidence and is likely to change the estimate; two of the domains were not fulfilled in the GRADE classification; and (4) very low quality—we are uncertain about the estimate; three of the domains in the GRADE classification were not fulfilled.^60 61^

### Statistical analysis

Clinical heterogeneity was assessed through visual examination of the data extraction table on details related to participant characteristics, intervention, study design and process in the included studies. Based on this assessment, the reviewers judged there to be low clinical heterogeneity and accordingly it was appropriate to perform a meta-analysis where feasible. The primary outcome was a measure of pain, disability or function. As pain scores were reported on different scales, we used the standardised mean difference (SMD).^62^ We a priori defined effect size interpretation as 0.2 for a ‘small’ effect size, 0.5 for a ‘medium’ effect size and 0.8 for a ‘large’ effect size, as suggested by Cohen (1988).^63^ If data were not available, the associated corresponding author was contacted. Failing this, the mean and SD were estimated, assuming normal distribution, from medians and IQRs.^64^ Statistical between-study heterogeneity was assessed with the I^2^^2^ statistic. We considered 0%–25% as low, 26%–74% moderate and 75% and over as high statistical heterogeneity.^65^ When outcomes presented with low statistical heterogeneity, data were pooled using a fixed-effects model.^66^ When analyses presented with moderate or high statistical heterogeneity, a DerSimonian and Laird random-effects model was adopted.^67^

All data analyses were performed using the OpenMetaAnalyst software.^68^

### Sensitivity analysis

A sensitivity analysis was performed for the primary and secondary analyses using only trials that presented with a low risk of bias.^56^ In addition we carried out a sensitivity analysis to assess the impact of studies where mean and SD were estimated from medians and IQRs, and outcome measures of pain were pooled scores set within pain domains from patient-reported outcome measures, for example, the Shoulder Pain and Disability Index (SPADI).^69^

## Results

### Study identification

**Figure 1:**
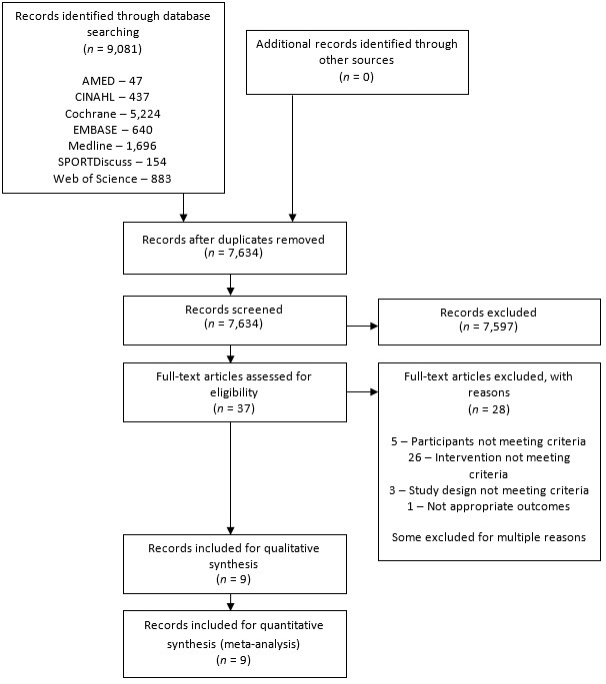
PRISMA 2009 flow diagram.

The search results are presented in figure 1. The database search produced 9081 results, with no additional findings from reference list searches or unpublished searches. After duplicates were removed, 37 papers were appropriate for full-text review.

After full-text review, 28 articles were excluded, 5 were due to participants not meeting the criteria, 26 because the intervention did not meet the criteria, 3 because of study design not meeting criteria, and 1 due to inappropriate outcome measures. Some articles were excluded for multiple reasons. Therefore nine articles were included in the final review. Of the included articles, there were two occurrences of the same trial reporting different time points over two publications.^43 70–72^

### Characteristics of included trials

A summary of the characteristics and main findings of the included trials can be found in table 2.

**Table 2:** Characteristics of included trials

**Study characteristics**	**Participant characteristics**	**Intervention and setting**	**Outcome data/results**
Aasa *et al* (2015)^43^ Michaelson *et al* (2016)^72^ 2 groups: 1. High-load lifting exercise 2. Low-load motor control exercises	70 patients recruited from occupational healthcare services in Sweden (mean age 42, 56% female); inclusion criteria included: (a) adults with low back pain >3 months’ duration and (b) with or without leg pain	Physiotherapy clinic, sports centre and home setting 1. n=35; group exercises based at a sports centre (5 participants in each group), with pain up to 50 mm Visual Analogue Scale acceptable, such that the pain subsided after each set of exercises; 12 treatment sessions over an 8-week period (weeks 1–4, 2 sessions per week; weeks 5–8, 1 session per week); 60 min in duration; no home exercises 2. n=35; pain-free individual exercises at a physiotherapy centre; 12 treatment sessions over an 8-week period (weeks 1–4, 2 sessions per week; weeks 5–8, 1 session per week); 20–30 min in duration; exercises involved improving control around joint neutral positions; in supine, four-point kneeling, sitting, and/or standing positions; Plus home exercises, 10 repetitions 2–3× a day	Main outcome assessed at baseline, 2-month and 12-month follow-up was 7 day average pain on a Visual Analogue Scale (0–100 mm) and Roland-Morris Disability Questionnaire (0–24) Group 1: mean pain at baseline 43 (SD 24), 2 months 22 (SD 21), 12 months 24 (SD 27) and 24 months 27 (SD 27) Group 2: mean pain at baseline 47 (SD 28), 2 months 30 (SD 26), 12 months 25 (SD 22) and 24 months 30 (SD 29) Group 1: mean disability at baseline 7.2 (SD 4.3), 2 months 3.8 (SD 4.0), 12 months 3.6 (SD 4.2) and 24 months 3.8 (SD 3.9) Group 2: mean disability at baseline 7.1 (SD 3.9), 2 months 3.6 (SD 4.2), 12 months 3.3 (SD 3.6) and 24 months 3.6 (SD 3.7) Both groups had significant improvements in their pain and disability levels; no significant between-group difference for pain at any follow-up (2 months p=0.71; 12 months p=0.94; 24 months p=0.89); no significant between-group difference for disability at any follow-up (2 months p=0.77; 12 months p=0.74; 24 months p=0.99)
Holmgren *et al* (2012)^70^ Hallgren *et al* (2014)^71^ 2 groups: 1. Specific exercises group 2. Control exercise group Patients were given the option at 3 months of continuing to have an arthroscopic subacromial decompression.	97 patients recruited from the waiting list for an arthroscopic subacromial decompression from a university hospital in Sweden (mean age 52, 37% female); inclusion criteria included (a) adults with lateral shoulder pain >6 months, (b) failed 3 months of previous primary care, (c) signs of impingement symptoms and (d) positive Neer’s impingement test of a subacromial anaesthetic injection	Physiotherapy and home setting 1. n=51; eccentric rotator cuff exercises and concentric/eccentric scapula exercises; recommendation of 5/10 numerical rating scale for pain during exercises, such that the pain subsided by the next exercise session; 7 physiotherapy appointment, weekly first 2 weeks, alternative weeks thereafter; exercises to be performed at home once or twice a day for 12 weeks 2. n=46; pain-free upper limb and neck exercises; 7 physiotherapy appointment, weekly first 2 weeks, alternative weeks thereafter; exercises to be performed at home once or twice a day for 12 weeks	Main outcome of Constant-Murley score (C-M) (0–100), along with shoulder assessment scores and pain scores taken at baseline, 3 months and 12 months, including pain at rest measured on Visual Analogue Scale (0–100 mm) Group 1: mean C-M at baseline 48 (SD 15), 3 months 72 (SD 19) and 12 months 83 (SD 14) Group 2: mean C-M at baseline 43 (SD 15), 3 months 52 (SD 23) and 12 months 76 (SD 18) Group 1: mean pain at rest at baseline 15 (SD 19), 3 months 10 (SD 14) and 12 months 2 (SD 6) Group 2: mean pain at rest at baseline 20 (SD 21), 3 months 20 (SD 25) and 12 months 4 (SD 13) Both groups had significant improvements in all outcomes at 3 months and 1 year follow-up. Significantly more patients in the control group decided to have surgery (63%) than those in the specific exercise group (24%; p<0.0001).
Littlewood *et al* (2015)^52^ 2 groups: 1. Self-managed exercises 2. Usual physiotherapy	86 patients recruited from UK, NHS physiotherapy waiting list (mean age 55, 50% female); inclusion criteria included (a) adults with shoulder pain >3 months, (b) maintained shoulder ROM and (c) pain with resisted movements	Physiotherapy and home setting 1. n=42; single shoulder exercise guided by the symptomatic response, requiring pain to be produced during exercise, such that the pain subsided after the exercises; typically involving a weighted shoulder abduction exercise of 3 sets of 10–15 repetitions; pragmatic approach to number of follow-ups, timings of appointments and point of discharge; that is, the treating physiotherapist and patient will determine these factors 2. n=44; usual physiotherapy,* including advice, stretching, exercise, manual therapy, massage, strapping, acupuncture, electrotherapy, corticosteroid injection at the discretion of the treating physiotherapist; pragmatic approach to number of follow-ups, timings of appointments and point of discharge; that is, the treating physiotherapist and patient will determine these factors	Main outcome of the Shoulder Pain and Disability Index (SPADI) (0–100) at baseline, 3, 6 and 12 months Group 1: mean at baseline 49.1 (SD 18.3), 3 months 32.4 (SD 20.2), 6 months 16.6 (SD 19.7) and 12 months 14.2 (SD 20.0) Group 2: mean at baseline 49.0 (SD 18.0), 3 months 30.7 (SD 19.7), 6 months 24.0 (SD 19.7) and 12 months 21.4 (SD 25.4) Statistically significant and clinically important within group changes for SPADI from baseline to all three follow-up points. There were no statistically significant differences between the groups across all the outcomes at 3, 6 or 12 months, (p=0.75, 0.19 and 0.32, respectively).
Maenhout *et al* (2013)^47^ 2 groups: 1. Traditional rotator cuff training with heavy load eccentric training 2. Traditional rotator cuff training	61 patients recruited from a shoulder surgeon’s clinic in Belgium (mean age 39.8, 41% female); inclusion criteria included (a) adults with >3 months of shoulder pain, (b) painful arc, (c) 2 out of 3 impingement tests, (d) pain on palpation of rotator cuff tendons	Physiotherapy and home setting 1. n=31; the same exercises as group 2, plus a heavy loaded eccentric exercise of abduction within the scapular plane; 3 sets of 15 repetitions, such that the patient experiences pain on the last set, up to 5/10 Visual Analogue Scale, such that the pain subsided by the following morning. 2. n=30; pain-free, traditional rotator cuff exercises of internal and external rotation with a resisted rubber band; performed once a day, with 3 sets of 10 repetitions; both groups had exercise prescription and monitoring through 9 physiotherapy appointments over 12 weeks	Main outcome of the SPADI (0–100) at baseline, 6 weeks and 12 weeks Group 1: mean at baseline 44.3 (SD 11.5), 6 weeks 17.7 (SD 12.0) and 12 weeks 14.5 (SD 11.7). Group 2: mean at baseline 42.0 (SD 11.0), 6 weeks 25.4 (SD 11.9) and 12 weeks 17.0 (SD 11.4) In both groups pain and function, measured with the SPADI score, improved significantly over time (p>0.001). When comparing between groups, improvement of the SPADI score was not significantly different.
Nørregaard *et al* (2007)^73^ 2 groups: 1. Eccentric exercises 2. Stretching exercises	45 patients recruited from a clinic of sports medicine in Denmark (mean age 42, 49% female); inclusion criteria included (a) adults with Achilles pain >3 months, (b) local thickening >2 mm on ultrasound, (c) diffuse posterior ankle pain	Sports medicine clinic and home setting 1. n=21; information leaflet with home exercise programme on; to be performed twice a day, for 12 weeks; 1 follow-up appointment at 3 months; 3 sets of 15 repetitions of eccentric calf exercises, with knee straight and semi-flexed; patients told to expect pain during the exercises, but to avoid increasing daily pain or morning stiffness 2. n=24; information leaflet with home exercise programme on; to be performed twice a day, for 12 weeks; 1 follow-up appointment at 3 months; pain-free standing stretches for gastrocnemius and soleus; 5 repetitions of 30 s each	Outcome measures were tenderness on palpation, ultrasound and pain, as measured by the Knee Injury and Osteoarthritis Outcome Score (KOOS) (0–4) and patient’s global assessment; follow-up was at baseline, 3, 6, 9, 12 weeks and 1 year Group 1: mean pain domain from KOOS at baseline 1.6 (SD 0.6), 3 weeks 0.1 (SD 0.1), 6 weeks 0.3 (SD 0.1), 9 weeks 0.4 (SD 0.2), 12 weeks 0.4 (SD 0.2) and 1 year score was 1.0 (SD 0.2) Group 2: mean pain domain from KOOS at baseline 1.6 (SD 0.6), 3 weeks 0.2 (SD 0.1), 6 weeks 0.3 (SD 0.1), 9 weeks 0.3 (SD 0.2), 12 weeks 0.4 (SD 0.2) and 1 year score was 0.7 (SD 0.2) There were significant improvements in all dimensions of the KOOS compared with baseline, with no differences between group differences.
Rathleff *et al* (2015)^51^ 2 groups: 1. High-load strengthening exercises 2. Stretching exercises	48 patients recruited from a university hospital, regional hospital and private clinic in Denmark (mean age 46, 66% female); inclusion criteria included (a) adults with plantar fasciitis >3 months, (b) pain on palpation, (c) local thickening >4 mm on ultrasound	Home based exercises 1. n=24; information leaflet, heel inserts and a prescription of a high-load strength programme; consisting of single calf raises with a towel rolled up under the toes for maximum toe extension, activating the windlass mechanism; each calf raises was 3 s up, 2 s pause, 3 s down; weight was added in rucksacks, starting at 12 repetition maximum for three sets, and slowly progressed over 3 months; patients were advised to perform the exercise every other day; exercises were allowed to be painful, with no postincrease in pain. 2. n=24; information leaflet, heel inserts and a prescription of pain-free* plantar-specific stretches; patients were asked to stretch the plantar fascia in a cross-legged position by extending their toes, hold for 10 s, 10 times, 3× a day for 3 months	Primary outcome was Foot Function Index at 1, 3, 6 and 12 months, including pain at worse and pain on first step on a numerical rating scale (0–10). Mean scores for group 1 pain at worse at baseline was 7.9 (SD 1.7), 1 month 6.1 (95% CI 5.1 to 7.2), 3 months 3.5 (95% CI 2.3 to 4.7), 6 months 2.5 (95% CI 1.4 to 3.6) and 12 months 2.9 (95% CI 1.7 to 4.0). Mean scores for group 2 pain at worse at baseline was 7.5 (SD 1.6), 1 month 6.1 (95% CI 5.2 to 7.1), 3 months 6.1 (95% CI 4.4 to 7.7), 6 months 3.4 (95% CI 2.0 to 4.7) and 12 months 1.8 (95% CI 0.7 to 3.0). At 3 months group 1 had significantly lower pain scores than group 2 (p<0.05). At months 1, 6 and 12, there was no significant difference between groups.
Silbernagel *et al* (2001)^74^ 2 groups: 1. Eccentric exercises 2. Regular concentric/eccentric exercises	40 patients recruited from mailings to hospitals, clinics and sports clubs in Sweden (mean age 45, 23% female); inclusion criteria included (a) adults with Achilles pain >3 months	Clinic and home setting 1. n*=*22; progressive eccentric exercise programme to be performed 2× a day, plus three sets of six different stretching exercises, 20 s each, as well as balance, toe/heel walking exercises; weekly physiotherapy contact for 12 weeks; pain was allowed during the exercises up to 5/10 Visual Analogue Scale, such that the pain subsided by the following morning with no morning stiffness 2. n=18. 3 x a day of regular concentric and eccentric calf strengthening, plus two sets of the stretching exercises from group 1. Physiotherapy contacts 3–5 x during the 12 weeks. Exercises must be pain-free.	Outcomes of pain on palpation (Visual Analogue Scale) (0–100 mm) taken at baseline, 6 weeks, 3 and 6 months. Other outcomes included pain on walking and pain on stairs (yes/no), various objective measures, plus a non-validated functional questionnaire Median±IQR scores for pain on palpation for group 1 at baseline was 49±26.2, 6 weeks 40±27.5, 3 months 35±24.8 and 6 months 21±20. Median±IQR scores for group 2 at baseline was 27±21.5, 6 weeks 20±20, 3 months 31±26 and 6 months 9±17.5. There was a significant decrease in pain on palpation in both treatment groups; no significant differences between groups were seen.

*Information not within publication, authors contacted for clarification.

ROM, range of motion.

The two occurrences of the same trial reporting different time points over two articles were analysed as single trials to prevent multiplicity in analyses.^43 70–72^ All trials investigated home-based exercises, had a roughly even composition of women and men (46% women), with similar mean ages of participants (mean age 47, range 19–83). One trial included low back pain,^43 72^ three included shoulder pain,^47 52 70 71^ two included Achilles pain^73 74^ and one included plantar heel pain.^51^

Three trials used a Visual Analogue Scale to measure pain,^43 70–72 74^ two trials used the SPADI,^47 52^ one used the Knee Injury and Osteoarthritis Outcome Score (KOOS),^73^ and one used the Foot Function Index (FFI) including pain at worse and pain on first step on a numerical rating scale (0–10).^51^

Where pain outcomes were included within patient-reported outcome measures, these data were extracted.^47 52 73^ Two trials that used the SPADI had insufficient data in the publication to complete a meta-analysis for pain,^47 52^ and both were contacted and asked to supply pain domain data. Littlewood *et al*^52^ replied and provided all the available data; however, Maenhout *et al*^47^ did not respond. One trial reported outcomes in medians and IQRs,^74^ and was contacted and asked for further data. They were unable to supply this, so the mean and SD were estimated assuming normal distribution.^64^

All seven trials recorded short-term follow-up of pain, four trials recorded medium-term follow-up of pain,^47 51 52 74^ and five trials recorded long-term follow-up for pain.^43 51 52 70–73^

### Trial quality and bias

The two papers reporting long-term outcomes for the trials that reported different time points made reference to the short-term outcome papers with regard to design parameters; therefore, trial quality and bias were assessed accordingly.^43 70–72^

**Figure 2:**
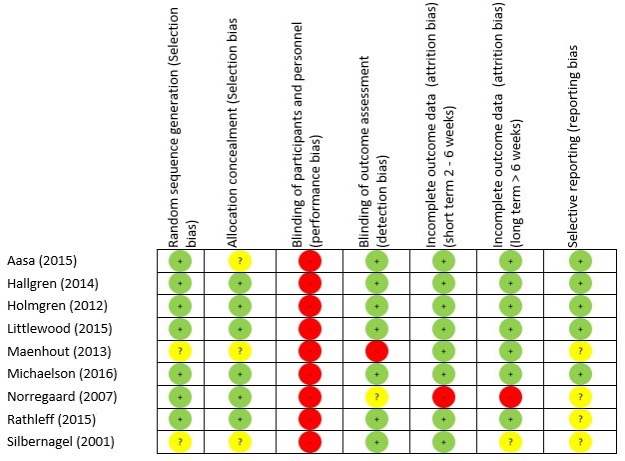
Risk of bias summary.

No trial had greater than three ‘high risk’ of bias scores for a domain (figure 2).

**Figure 3:**
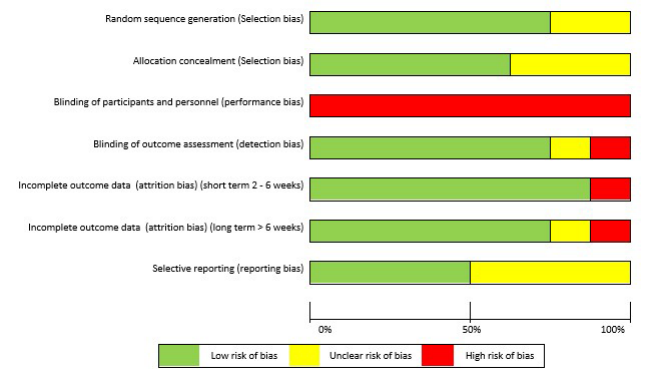
Risk of bias graph.

The greatest risk of bias was with the blinding of participants and personnel (100%) (figure 3). The greatest amount of uncertainty was with regard to selective reporting bias, as many of the trials failed to include trials register details, or protocol details (44%).^47 51 73 74^ Other common areas of bias with the included trials were with attrition bias, one trial failed to adequately describe attrition,^43^ and two trials had large dropout rates^52 73^; however, Littlewood *et al*^52^ received a ‘low risk’ score as their participant attrition was balanced across the intervention and control groups,^75^ and an intention-to-treat analysis was performed. The risk of bias assessment tool highlights common trial write-up errors, with a number of papers failing to give an appropriate level of detail to adequately assess selection bias risk (33%).^43 47 74^

### Narrative synthesis of disability and function outcomes

Of the seven trials, six reported some form of patient-reported outcome measure of disability or function. One reported Roland-Morris Disability Questionnaire,^43 72^ one reported Constant-Murley and the Disabilities of the Arm Shoulder and Hand score,^70 71^ two reported the SPADI,^47 52^ one reported the KOOS,^73^ and one reported the FFI.^51^ With the exception of Rathleff *et al,*^51^ there was clinically significant improvements in all outcomes, with no clear superiority. At 3-month follow-up for Rathleff *et al,*^51^ the intervention group had a statistically significant lower FFI than the control group (p=0.016). At 1, 6 and 12 months, there were no differences between groups (p>0.34).

### Contextual factors

With regard to the parameters of pain in the exercise intervention the participants were advised to adhere to, each trial gave different instructions, the key differences being if pain was allowed^43 51 72 74^ or recommended.^47 52 70 71 73^ In addition other differences were if an acceptable level of pain measured on a pain scale was advised,^47 70 71 74^ and a time frame for the pain to subside by, for instance, if the pain had to subside immediately,^43 51 52 72^ by the next session^70 71^ or by the next day.^47 73 74^ Clinically significant improvements in patient-reported outcome measures were reported across all interventions and control exercises, and all time points. It is not clear from the data if one approach was superior to the others.

### Meta-analysis of pain

#### Short-term results

**Figure 4:**
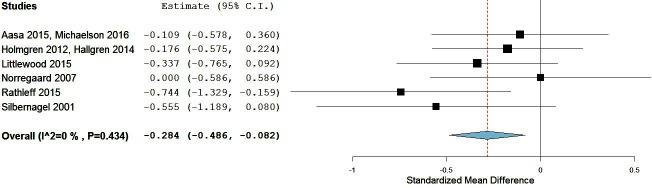
Forest plot of exercises into pain versus pain-free exercises—short term. Negative values favour painful intervention, whereas positive favour pain-free.

Six trials with 385 participants reported post-treatment effect on pain. Combining the results of these trials demonstrated significant benefit (SMD) of exercises into pain compared with pain-free exercises for musculoskeletal pain in the short term, with a small effect size of −0.28 (95% CI −0.49 to −0.08; figure 4). Statistical heterogeneity was negligible, I^2^=0%. The quality of evidence (GRADE) was rated as ‘low quality’ due to trial design and low participant numbers (table 3).

**Table 3:** GRADE summary of findings table

**Summary of results**				**Quality of the evidence (GRADE)**			
**Follow-up**	**Number of participants(trials)**	**SMD** **(95% CI)**	**Design**	**Inconsistency**	**Indirectness**	**Imprecision**	**Quality**
Short term	385 (6 trials)	−0.28 (−0.49 to −0.08)	Limitations*	No inconsistency	No indirectness	Imprecision†	**Low** ⨁⨁◯◯
Medium term	173 (3 trials)	−0.59 (−1.03 to −0.15)	Limitations*	No inconsistency	No indirectness	Imprecision†	**Low** ⨁⨁◯◯
Long term	345 (5 trials)	0.01 (−0.39 to 0.41)	Limitations*	Inconsistency‡	No indirectness	Imprecision†	**Very low** ⨁◯◯◯
		**Sensitivity analysis**					
Short term	215 (3 trials)	−0.27 (−0.54 to −0.05)	No limitations	No inconsistency	No indirectness	Imprecision†	**Moderate** ⨁⨁⨁◯
Medium term	40 (1 trials)	−0.32 (−0.95 to 0.31)	No limitations	Inconsistency§	No indirectness	Imprecision†	**Low** ⨁⨁◯◯
Long term	215 (3 trials)	0.13 (−0.14 to 0.40)	No limitations	No inconsistency	No indirectness	Imprecision†	**Moderate** ⨁⨁⨁◯

*Lack of blinding of participants and personnel, attrition bias, unable to adequately assess selection bias risk.

†<400 participants for each outcome.

‡Large statistical heterogeneity; I^2^=70%.

§Only single trial available, <400 participants therefore downgraded for inconsistency and imprecision.

Short term, ≤3 months; medium term, >3 and <12 months; long term, ≥12 months.

High quality: further research is unlikely to change our confidence in the estimate of effect.

Moderate quality: further research is likely to have an important impact on our confidence in the estimate of effect.

Low quality: further research is very likely to have an important impact on our confidence in the estimate of effect.

Very low quality: we are uncertain about the estimate.

GRADE, Grading of Recommendations Assessment, Development and Evaluation; SMD, standardised mean difference.

For sensitivity analysis in the short term, we repeated the meta-analysis, removing two trials that used a patient-reported outcome measures index and had high dropout rates,^52 73^ and the Silbernagel *et al*^74^ trial where the mean and SD were estimated from medians and IQRs. The results of the data synthesis produced very similar results, with a small effect size of −0.27 (95% CI −0.54 to −0.05), with low statistical heterogeneity of I^2^=22%. The quality of evidence (GRADE) was rated as ‘moderate quality’ due to low participant numbers (table 3).

#### Medium-term results

**Figure 5:**
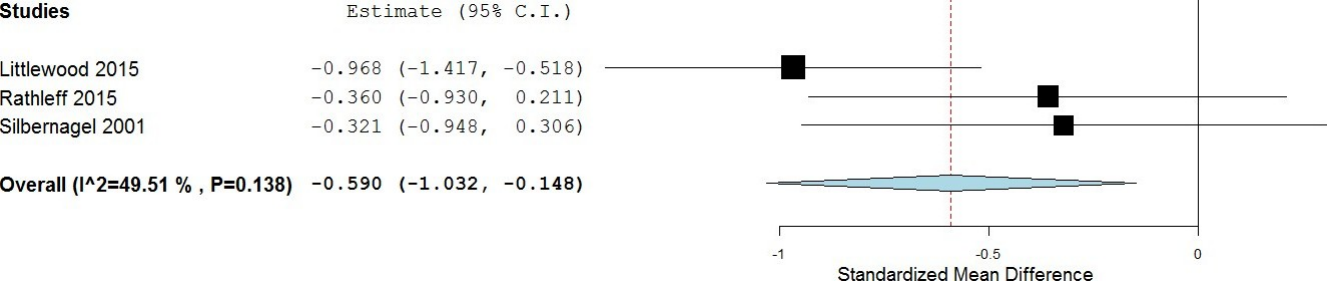
Forest plot of exercises into pain versus pain-free exercises—medium term. Negative values favour painful intervention, whereas positive favour pain-free.

In the medium-term follow-up, meta-analysis demonstrated significant benefit (SMD) for exercises into pain compared with pain-free exercises for musculoskeletal pain, with a medium effect size of −0.59 (95% CI −1.03 to −0.15) (see figure 5). The statistical heterogeneity was moderate, I^2^=50%. The quality of evidence (GRADE) was rated as ‘low quality’ due to trial design and low participant numbers (table 3).

Sensitivity analysis was not possible for medium-term results as two trials were excluded, one for using a patient-reported outcome measures index,^51^ and one due to means and SD being estimated from medians and IQRs.^74^ The one remaining trial showed no significant difference in the medium term.^51^ The quality of evidence (GRADE) was rated as ‘low quality’ due to it being only from a single trial (table 3).

#### Long-term results

**Figure 6:**
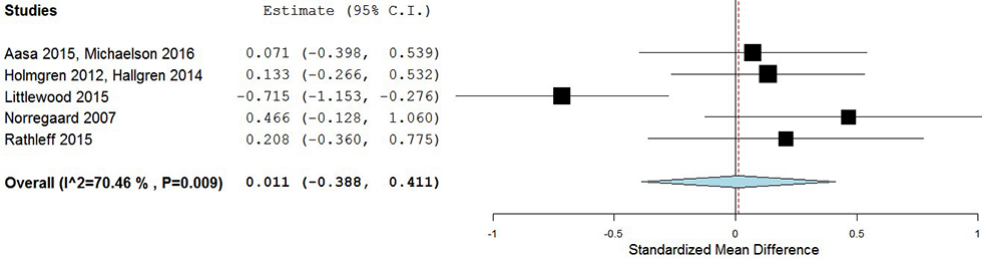
Forest plot of exercises into pain versus pain-free exercises—long term. Negative values favour painful intervention, whereas positive favour pain-free. AMED, Allied and Complimentary Medicine Database; CINAHL, Cumulative Index to Nursing and Allied Health Literature.

In the long term follow-up, meta-analysis demonstrated no statistical difference between exercises into pain and pain-free exercises, with an effect size of 0.01 (95% CI −0.39 to 0.41) (figure 6). The statistical heterogeneity was high, I^2^=70%. The quality of evidence (GRADE) was rated as ‘very low quality’ due to trial design, heterogeneity and low participant numbers (table 3).

For sensitivity analysis in the long term, we repeated the meta-analysis, removing the two trials that used a patient-reported outcome measures index.^52 73^ The results of the data synthesis found no statistical difference between exercises into pain and pain-free exercises, with an effect size of 0.13 (95% CI −0.14 to 0.40). The statistical heterogeneity was negligible, I^2^=0%. The quality of evidence (GRADE) was rated as ‘moderate quality’ due to low participant numbers (table 3).

## Discussion

### Summary of main findings

There was a significant short-term benefit for exercises into pain over pain-free exercises for patient-reported outcomes of pain, with a small effect size and moderate quality of evidence. There appears to be no difference at medium-term or long term follow-up, with the quality of the evidence rated as moderate to low.

### Clinical and research implications

Traditionally, healthcare practitioners have been reluctant to encourage patients to continue with exercise into pain when they are treating chronic musculoskeletal pain,^76^ with some research suggesting clinicians’ fear being the primary deterrent.^77^ The results of our systematic review show that there does not appear to be a scientific basis for this fear in relation to outcome measures of pain, and also potentially function and disability. This is an important point when considering what advice is given on any short-term exacerbations of musculoskeletal pain during physical activity or exercise by healthcare practitioners, particularly when physical inactivity is one of the 10 leading risk factors for death worldwide,^78^ and when an estimated €1.9 billion a year in healthcare and €9.4 billion a year in economic costs in the UK are attributable to physical inactivity.^79^

A theoretical rationale for a positive response to exercises into pain is the positive impact on the central nervous system.^31 37^ Specifically, the exercise addresses psychological factors such as fear avoidance, kinesiophobia and catastrophising, and is set within a framework of ‘hurt not equalling harm’, thus, in time, reducing the overall sensitivity on the central nervous system, with a modified pain output.^31 37^ The exercise-induced endogenous analgesia effect is thought to occur due to a release of endogenous opioids and activation of spinal inhibitory mechanisms.^80–84^ However, a recent systematic review has established that no firm conclusions could be reached about pain modulation during exercise therapy for chronic musculoskeletal pain.^85^ Indeed one experimental study has shown a dysfunction of endogenous analgesia in patients with musculoskeletal pain,^86^ and therefore exercising non-painful body parts with patients with chronic musculoskeletal pain has been recommended.^87^ However, it is worth noting that empirical data within this field are greatly lacking, and this systematic review shows that painful exercises may even improve the clinical outcomes. Additionally, exercise prescription in the included trials was primarily based on strength and conditioning principles, with the exception of Littlewood *et al*,^52^ suggesting a tissue-focused approach, and therefore could still have been giving a ‘hurt is harm’ message to the majority of participants.

Significant improvements in patient-reported pain can be achieved with a range of contextual factors, such as varying degrees of pain experiences and postrecovery time for therapeutic exercise. In addition to the aspect of pain, an important difference between the intervention arm and the control arm is the higher loads, or levels of resistance, employed with the exercises into pain, and it is unknown if the difference in responses can be attributable to these two elements of the different exercise programmes. Research has shown a ‘dose response’ to exercise for musculoskeletal pain—the more incremental exercise (with appropriate recovery period) a person does the greater his/her improvements in pain^88–90^; the short-term benefits of exercises into pain over pain-free exercises could be explained by this dose effect, or response to load/resistance. However to our knowledge the optimal ‘dose’ of therapeutic exercise for musculoskeletal pain has not been established. Furthermore, little is known if it is possible or appropriate to identify individuals most suitable to exercise interventions.

Our review only investigated patient-reported outcome measures of pain and function/disability. It has been hypothesised that exercise therapy, where it has been advised that the experience of pain is safe and allowed, may address other patient-reported outcome measures—fear avoidance, self-efficacy and catastrophising beliefs^37 38^—and therefore may lead to improvements in function, quality of life and disability, despite pain levels. Unfortunately none of the trials included in this review recorded the level of pain patients actually experienced during their exercise programme, preventing any detailed attempt to fully explain any mechanisms of effect. This aspect of exercise prescription clearly warrants further investigation in relation to chronic musculoskeletal pain. Any future trials should consider the role of pain with exercises and clearly define the parameters employed to ensure translation of findings into practice and further evaluation of optimal ‘dosage’.

### Strengths and limitations of included trials

We chose not to perform subgroup analyses by anatomical region and/or tissue structures. The labelling of musculoskeletal structures as sources of pain has been debated for many years, with polarising opinions.^91 92^ However, the diagnostic labelling of patients into tissue-specific pathology characteristically suffers from poor reliability and validity.^93–98^ A strength of this review is that despite the trials including subjects suffering from musculoskeletal pain at different body locations, there exists low statistical heterogeneity at short-term follow-up and for the sensitivity analyses carried out.

The overall quality of the included papers can be considered relativity high, with only three domains in the Cochrane risk of bias tool (disregarding blinding of participants) demonstrating clear risk of bias across all domains for all trials. However taking into account other factors assessed with the GRADE analysis, the quality of the evidence was rated as moderate to low. Therefore our results can be considered to have moderate to low internal validity, with future research likely to alter our conclusions.

The main source of bias within the included trials were blinding; no trial blinded the participants. Knowledge of group assignment may affect participants’ behaviour, for example with patient-reported outcome measures such as pain scales or compliance with therapy interventions.^99^ However, it is accepted that blinding in physiotherapy and physical intervention trials is difficult to achieve.^24^

Another limitation of the included trials is the high level of attrition suffered by some of the trials in both treatment arms. For example Littlewood *et al*^52^ suffered from 51% dropout at 12-month follow-up. A high level of attrition can overestimate the treatment effect size and could bias the results of our meta-analysis. However, we minimised the risk of bias on our results by conducting a sensitivity analysis on trials with a large dropout, identified using the Cochrane risk of bias tool and assessed level of evidence using the GRADE classification.

### Limitations of this review

For pragmatic reasons one reviewer screened titles and abstracts. An extensive literature search was carried out, with two reviewers independently screening full texts for inclusion, and a sample of the data extraction independently verified. Additionally an attempt was made to retrieve unpublished trials; however, it may be that not all trials were retrieved, particularly considering we did not search for papers published in languages other than English and US spelling was used in the search terms. This review excluded trials where participants had a diagnosis of more widespread pain disorders like fibromyalgia.

## Conclusion

The results of this systematic review indicates that protocols using exercises into pain offer a small but significant benefit over pain-free exercises in the short term, with moderate quality of the evidence for outcomes of pain in chronic musculoskeletal pain in adults. There appears to be no difference at medium-term or long-term follow-up, with moderate to low quality of evidence, demonstrating pain need not be ruled out or avoided in adults with chronic musculoskeletal pain.

**What are the findings?**

- Protocols using exercises into pain for chronic musculoskeletal pain offer a small but significant benefit over pain-free exercises in the short term.
- Adults with musculoskeletal pain can achieve significant improvements in patient-reported outcomes with varying degrees of pain experiences and postrecovery time with therapeutic exercise.
- Pain during therapeutic exercise for chronic musculoskeletal pain need not be a barrier to successful outcomes.
- Protocols using exercises into pain typically have higher loads and dose of exercise.

## References

[1] Lawrence RC, Helmick CG, Arnett FC, et al Estimates of the prevalence of arthritis and selected musculoskeletal disorders in the United States. Arthritis Rheum 1998;41:778–99. 10.1002/1529-0131(199805)41:5<778::AID-ART4>3.0.CO;2-V 9588729

[2] Murray CJ, Vos T, Lozano R, et al Disability-adjusted life years (DALYs) for 291 diseases and injuries in 21 regions, 1990-2010: a systematic analysis for the global burden of disease study 2010. Lancet 2012;380:2197–223. 10.1016/S0140-6736(12)61689-4 23245608

[3] Hoy D, March L, Brooks P, et al The global burden of low back pain: estimates from the global burden of disease 2010 study. Ann Rheum Dis 2014;73:968–74. 10.1136/annrheumdis-2013-204428 24665116

[4] Vos T, Flaxman AD, Naghavi M, et al Years lived with disability (YLDs) for 1160 sequelae of 289 diseases and injuries 1990-2010: a systematic analysis for the global burden of disease study 2010. Lancet 2012;380:2163–96. 10.1016/S0140-6736(12)61729-2 23245607 PMC6350784

[5] Hoy D, March L, Woolf A, et al The global burden of neck pain: estimates from the global burden of disease 2010 study. Ann Rheum Dis 2014;73:1309–15. 10.1136/annrheumdis-2013-204431 24482302

[6] Smith E, Hoy DG, Cross M, et al The global burden of other musculoskeletal disorders: estimates from the global burden of disease 2010 study. Ann Rheum Dis 2014;73:1462–9. 10.1136/annrheumdis-2013-204680 24590181

[7] *Department of Health*. The musculoskeletal services framework- A joint responsibilty: doing itdifferently–. London: DH Publications Orderline, 2006:1–72.

[8] HSE. Work-related musculoskeletal disorder statistics, great britain 2016. 2016:1–20 www.hse.gov.uk/statistics.

[9] Mason L, Moore RA, Edwards JE, et al Topical NSAIDs for chronic musculoskeletal pain: systematic review and meta-analysis. BMC Musculoskelet Disord 2004;5:28 10.1186/1471-2474-5-28 15317652 PMC516039

[10] Noble M, Treadwell JR, Tregear SJ, et al Long-term opioid management for chronic noncancer pain. Cochrane Database Syst Rev 2010;1. 10.1002/14651858.CD006605.pub2 PMC6494200 20091598

[11] Roelofs PD, Deyo RA, Koes BW, et al Non-steroidal anti-inflammatory drugs for low back pain. Cochrane Database Syst Rev 2008 1:CD000396 10.1002/14651858.CD000396.pub3 PMC10220428 18253976

[12] Chou R, Huffman LH Medications for acute and chronic low back pain: a review of the evidence for an american Pain Society/American College of Physicians clinical practice guideline. Ann Intern Med 2007;147:505–14. 10.7326/0003-4819-147-7-200710020-00008 17909211

[13] Ravenek MJ, Hughes ID, Ivanovich N, et al A systematic review of multidisciplinary outcomes in the management of chronic low back pain. Work 2010;35:349–67. 10.3233/WOR-2010-0995 20364056

[14] Scascighini L, Toma V, Dober-Spielmann S, et al Multidisciplinary treatment for chronic pain: a systematic review of interventions and outcomes. Rheumatology 2008;47:670–8. 10.1093/rheumatology/ken021 18375406

[15] van Geen JW, Edelaar MJ, Janssen M, et al The long-term effect of multidisciplinary back training: a systematic review. Spine 2007;32:249–55. 10.1097/01.brs.0000251745.00674.08 17224822

[16] van Middelkoop M, Rubinstein SM, Kuijpers T, et al A systematic review on the effectiveness of physical and rehabilitation interventions for chronic non-specific low back pain. Eur Spine J 2011;20:19–39. 10.1007/s00586-010-1518-3 20640863 PMC3036018

[17] Rubinstein SM, van Middelkoop M, Assendelft WJ, et al Spinal manipulative therapy for chronic low-back pain: an update of a Cochrane review. Spine 2011;36:E825–46. 10.1097/BRS.0b013e3182197fe1 21593658

[18] Miller J, Gross A, D’Sylva J, et al Manual therapy and exercise for neck pain: a systematic review. Man Ther 2010;15:334–54. 10.1016/j.math.2010.02.007 20593537

[19] Furlan AD, Imamura M, Dryden T, et al Massage for low‐back pain. Cochrane Database Syst Rev 2008: (4):CD001929 10.1002/14651858.CD001929.pub2 18843627

[20] Hall A, Maher C, Latimer J, et al The effectiveness of Tai Chi for chronic musculoskeletal pain conditions: a systematic review and meta-analysis. Arthritis Rheum 2009;61:717–24. 10.1002/art.24515 19479696

[21] Slade SC, Keating JL Unloaded movement facilitation exercise compared to no exercise or alternative therapy on outcomes for people with nonspecific chronic low back pain: a systematic review. J Manipulative Physiol Ther 2007;30:301–11. 10.1016/j.jmpt.2007.03.010 17509439

[22] Hendrick P, Te Wake AM, Tikkisetty AS, et al The effectiveness of walking as an intervention for low back pain: a systematic review. Eur Spine J 2010;19:1613–20. 10.1007/s00586-010-1412-z 20414688 PMC2989236

[23] Hall J, Swinkels A, Briddon J, et al Does aquatic exercise relieve pain in adults with neurologic or musculoskeletal disease? A systematic review and meta-analysis of randomized controlled trials. Arch Phys Med Rehabil 2008;89:873–83. 10.1016/j.apmr.2007.09.054 18452734

[24] Smith BE, Littlewood C, May S An update of stabilisation exercises for low back pain: a systematic review with meta-analysis. BMC Musculoskelet Disord 2014;15:416 10.1186/1471-2474-15-416 25488399 PMC4295260

[25] Miles CL, Pincus T, Carnes D, et al Can we identify how programmes aimed at promoting self-management in musculoskeletal pain work and who benefits? A systematic review of sub-group analysis within RCTs. Eur J Pain 2011;15 10.1016/j.ejpain.2011.01.016 21354838

[26] Moseley GL, Arntz A The context of a noxious stimulus affects the pain it evokes. Pain 2007;133:64–71. 10.1016/j.pain.2007.03.002 17449180

[27] Harvie DS, Broecker M, Smith RT, et al Bogus visual feedback alters onset of movement-evoked pain in people with neck pain. Psychol Sci 2015;26:385–92. 10.1177/0956797614563339 25691362

[28] Lobanov OV, Zeidan F, McHaffie JG, et al From cue to meaning: brain mechanisms supporting the construction of expectations of pain. Pain 2014;155:129–36. 10.1016/j.pain.2013.09.014 24055334 PMC3947355

[29] Nijs J, Lluch Girbés E, Lundberg M, et al Exercise therapy for chronic musculoskeletal pain: innovation by altering pain memories. Man Ther 2015;20:216–20. 10.1016/j.math.2014.07.004 25090974

[30] Meeus M, Nijs J, Van Wilgen P, et al Moving on to movement in patients with chronic joint pain. Pain Clin Updat 2016;24:1–8.

[31] Nijs J, Roussel N, Paul van Wilgen C, et al Thinking beyond muscles and joints: therapists' and patients' attitudes and beliefs regarding chronic musculoskeletal pain are key to applying effective treatment. Man Ther 2013;18:96–102. 10.1016/j.math.2012.11.001 23273516

[32] Zusman M Associative memory for movement-evoked chronic back pain and its extinction with musculoskeletal physiotherapy. Phys Ther Rev 2008;13:57–68. 10.1179/174328808X251948

[33] Zusman M Mechanisms of musculoskeletal physiotherapy. Phys Ther Rev 2004;9:39–49. 10.1179/108331904225003973

[34] Moseley GL Reconceptualising pain according to modern pain science. Phys Ther Rev 2007;12:169–78. 10.1179/108331907X223010

[35] Quartana PJ, Campbell CM, Edwards RR Pain catastrophizing: a critical review. Expert Rev Neurother 2009;9:745–58. 10.1586/ern.09.34 19402782 PMC2696024

[36] Moseley GL Joining forces – Combining Cognition-Targeted Motor Control Training with Group or Individual Pain Physiology Education: A Successful Treatment For Chronic Low Back Pain. J Man Manip Ther 2003;11:88–94. 10.1179/106698103790826383

[37] Littlewood C, Malliaras P, Bateman M, et al The central nervous system--an additional consideration in ’rotator cuff tendinopathy' and a potential basis for understanding response to loaded therapeutic exercise. Man Ther 2013;18:468–72. 10.1016/j.math.2013.07.005 23932100

[38] Smith BE, Hendrick P, Logan P Patellofemoral pain: challenging current practice - A case report. Man Ther 2016;22:216–9. 10.1016/j.math.2015.09.002 26394748

[39] Moher D, Liberati A, Tetzlaff J, et al Preferred reporting items for systematic reviews and Meta-Analyses: the PRISMA statement. PLoS Med 2009;6:e1000097 10.1371/journal.pmed.1000097 19621072 PMC2707599

[40] Cohen J A coefficient of agreement for nominal scales. Educ Psychol Meas 1960;20:37–46. 10.1177/001316446002000104

[41] Fleiss JL Statistical methods for rates and proportions. 2nd ed New York: John Wiley, 1981.

[42] Landis JR, Koch GG The measurement of observer agreement for categorical data. Biometrics 1977;33:159–74. 10.2307/2529310 843571

[43] Aasa B, Berglund L, Michaelson P, et al Individualized low-load motor control exercises and education versus a high-load lifting exercise and education to improve activity, pain intensity, and physical performance in patients with low back pain: a randomized controlled trial. J Orthop Sports Phys Ther 2015;45:77–85. 10.2519/jospt.2015.5021 25641309

[44] Beyer R, Kongsgaard M, Hougs Kjær B, et al Heavy Slow Resistance Versus Eccentric training as treatment for Achilles Tendinopathy. Am J Sports Med 2015;43:1704–11. 10.1177/0363546515584760 26018970

[45] Geraets JJ, Goossens ME, de Groot IJ, et al Effectiveness of a graded exercise therapy program for patients with chronic shoulder complaints. Aust J Physiother 2005;51:87–94. 10.1016/S0004-9514(05)70037-4 15924511

[46] Harts CC, Helmhout PH, de Bie RA, et al A high-intensity lumbar extensor strengthening program is little better than a low-intensity program or a waiting list control group for chronic low back pain: a randomised clinical trial. Aust J Physiother 2008;54:23–31. 10.1016/S0004-9514(08)70062-X 18298356

[47] Maenhout AG, Mahieu NN, De Muynck M, et al Does adding heavy load eccentric training to rehabilitation of patients with unilateral subacromial impingement result in better outcome? A randomized, clinical trial. Knee Surg Sports Traumatol Arthrosc 2013;21:1158–67. 10.1007/s00167-012-2012-8 22581193

[48] Østerås B, Østerås H, Torstensen TA, et al Dose-response effects of medical exercise therapy in patients with patellofemoral pain syndrome: a randomised controlled clinical trial. Physiotherapy 2013;99:126–31. 10.1016/j.physio.2012.05.009 23219636

[49] Schenk R, Dionne C, Simon C, et al Effectiveness of mechanical diagnosis and therapy in patients with back pain who meet a clinical prediction rule for spinal manipulation. J Man Manip Ther 2012;20:43–9. 10.1179/2042618611Y.0000000017 23372393 PMC3267446

[50] van der Plas A, de Jonge S, de Vos RJ, et al A 5-year follow-up study of Alfredson’s heel-drop exercise programme in chronic midportion Achilles tendinopathy. Br J Sports Med 2012;46:214–8. 10.1136/bjsports-2011-090035 22075719 PMC3277725

[51] Rathleff MS, Mølgaard CM, Fredberg U, et al High-load strength training improves outcome in patients with plantar fasciitis: a randomized controlled trial with 12-month follow-up. Scand J Med Sci Sports 2015;25:e292–300. 10.1111/sms.12313 25145882

[52] Littlewood C, Bateman M, Brown K, et al A self-managed single exercise programme versus usual physiotherapy treatment for rotator cuff tendinopathy: a randomised controlled trial (the SELF study). Clin Rehabil 2016;30:686–96. 10.1177/0269215515593784 26160149

[53] Østerås B, Østerås H, Torstensen TA, et al Long-term effects of medical exercise therapy in patients with patellofemoral pain syndrome: results from a single-blinded randomized controlled trial with 12 months follow-up. Physiotherapy 2013;99:311–6. 10.1016/j.physio.2013.04.001 23764516

[54] Higgins J, Deeks J Cochrane Handbook: General Methods For Cochrane Reviews: Ch 7: Selecting studies and collecting data : Higgins PTJ, Green S, Cochrane handbook for: systematic reviews of interventions. USA: Wiley-Blackwell, 2011:151–86.

[55] Furlan AD, Pennick V, Bombardier C, et al 2009 updated method guidelines for systematic reviews in the Cochrane Back Review Group. Spine 2009;34:1929–41. 10.1097/BRS.0b013e3181b1c99f 19680101

[56] Higgins JP, Altman DG, Gøtzsche PC, et al The Cochrane Collaboration’s tool for assessing risk of bias in randomised trials. BMJ 2011;343:d5928 10.1136/bmj.d5928 22008217 PMC3196245

[57] Clijsen R, Fuchs J, Taeymans J Effectiveness of exercise therapy in treatment of patients with patellofemoral pain syndrome: systematic review and meta-analysis. Phys Ther 2014;94:1697–708. 10.2522/ptj.20130310 25082920

[58] Atkins D, Best D, Briss PA, et al Grading quality of evidence and strength of recommendations. BMJ 2004;328:1490 10.1136/bmj.328.7454.1490 15205295 PMC428525

[59] Sterne JA, Egger M, Moher D Cochrane handbook: General methods for cochrane reviews: Ch 10: Addressing reporting biases : Higgins PTJ, Green S, Cochrane handbook for systematic reviews of interventions. USA: Wiley-Blackwell, 2011:297–334.

[60] Guyatt GH, Oxman AD, Vist GE, et al GRADE: an emerging consensus on rating quality of evidence and strength of recommendations. Chinese J. Evidence-Based Med 2009;9:8–11. 10.1136/bmj.39489.470347.AD PMC2335261 18436948

[61] Schünemann H, Brożek J, Guyatt G, et al GRADE handbook for grading quality of evidence and strength of recommendations. GRADE Work. Gr 2013.

[62] Hedges L V, Olkin I Statistical methods for meta-analysis. Phytochemistry 1985;72:369.

[63] Cohen J Statistical power analysis for the behavioral sciences. Stat Power Anal Behav Sci 1988 567.

[64] Hozo SP, Djulbegovic B, Hozo I, et al Estimating the mean and variance from the median, range, and the size of a sample. BMC Med Res Methodol 2005;5:13 10.1186/1471-2288-5-13 15840177 PMC1097734

[65] Higgins JP, Thompson SG, Deeks JJ, et al Measuring inconsistency in meta-analyses. BMJ 2003;327:557–60. 10.1136/bmj.327.7414.557 12958120 PMC192859

[66] Yusuf S, Peto R, Lewis J, et al Beta blockade during and after myocardial infarction: an overview of the randomized trials. Prog Cardiovasc Dis 1985;27:335–71. 10.1016/S0033-0620(85)80003-7 2858114

[67] DerSimonian R, Laird N Meta-analysis in clinical trials. Control Clin Trials 1986;7:177–88. 10.1016/0197-2456(86)90046-2 3802833

[68] Wallace BC, Dahabreh IJ, Trikalinos TA, et al Closing the gap between Methodologists and End-Users: r as a computational Back-End. Wiley Interdiscip Rev Comput 2012;49:1–15.

[69] Roach KE, Budiman-Mak E, Songsiridej N, et al Development of a shoulder pain and disability index. Arthritis Care Res 1991;4:143–9. 10.1002/art.1790040403 11188601

[70] Holmgren T, Björnsson Hallgren H, Öberg B, et al Effect of specific exercise strategy on need for surgery in patients with subacromial impingement syndrome: randomised controlled study. BMJ 2012;344:e787 10.1136/bmj.e787 22349588 PMC3282676

[71] Hallgren HC, Holmgren T, Oberg B, et al A specific exercise strategy reduced the need for surgery in subacromial pain patients. Br J Sports Med 2014;48:1431–6. 10.1136/bjsports-2013-093233 24970843

[72] Michaelson P, Holmberg D, Aasa B, et al High load lifting exercise and low load motor control exercises as interventions for patients with mechanical low back pain: a randomized controlled trial with 24-month follow-up. J Rehabil Med 2016;48:456–63. 10.2340/16501977-2091 27097785

[73] Nørregaard J, Larsen CC, Bieler T, et al Eccentric exercise in treatment of Achilles tendinopathy. Scand J Med Sci Sports 2007;17:133–8. 10.1111/j.1600-0838.2006.00545.x 17394474

[74] Silbernagel KG, Thomeé R, Thomeé P, et al Eccentric overload training for patients with chronic Achilles tendon pain--a randomised controlled study with reliability testing of the evaluation methods. Scand J Med Sci Sports 2001;11:197–206. 10.1034/j.1600-0838.2001.110402.x 11476424

[75] Higgins JPT, Altman DG, Jonathan AC Chapter 8: Assessing risk of bias in included studies In: Higgins JPT, Green S, eds Cochrane handbook for systematic reviews of interventions version 5.1.0 (updated march 2011). The cochrane collaboration, 2011, 2008 www.cochrane-www.handbook.org.

[76] Littlewood C, Lowe A, Moore J Rotator cuff disorders: a survey of Current UK physiotherapy practice. Shoulder Elbow 2012;4:64–71. 10.1111/j.1758-5740.2011.00164.x PMC5734529 29276538

[77] Littlewood C, Mawson S, May S, et al Understanding the barriers and enablers to implementation of a self-managed exercise intervention: a qualitative study. Physiotherapy 2015;101:279–85. 10.1016/j.physio.2015.01.001 25702093

[78] WHO. Global health risks: mortality and burden of disease attributable to selected Major risks. Bull World Health Organ 2009;87:646 10.2471/BLT.09.070565 19784438 PMC2739926

[79] ISCA/Cebr. The economic cost of physical inactivity in Europe. ISCA / Cebr Rep Published Online First: 2015.

[80] Koltyn KF Exercise-induced hypoalgesia and intensity of exercise. Sports Med 2002;32:477–87. 10.2165/00007256-200232080-00001 12076175

[81] Koltyn KF Analgesia following exercise: a review. Sports Med 2000;29:85–98. 10701712 10.2165/00007256-200029020-00002

[82] Koltyn KF, Arbogast RW Perception of pain after resistance exercise. Br J Sports Med 1998;32:20–4. 10.1136/bjsm.32.1.20 9562159 PMC1756063

[83] Ray CA, Carter JR Central modulation of exercise-induced muscle pain in humans. J Physiol 2007;585:287–94. 10.1113/jphysiol.2007.140509 17932155 PMC2375467

[84] Millan MJ Descending control of pain. Prog Neurobiol 2002;66:355–474. 10.1016/S0301-0082(02)00009-6 12034378

[85] Fuentes C JP, Armijo-Olivo S, Magee DJ, et al Effects of exercise therapy on endogenous pain-relieving peptides in musculoskeletal pain: a systematic review. Clin J Pain 2011;27:365–74. 10.1097/AJP.0b013e31820d99c8 21430521

[86] Van Oosterwijck J, Nijs J, Meeus M, et al Lack of endogenous pain inhibition during exercise in people with chronic whiplash associated disorders: an experimental study. J Pain 2012;13:242–54. 10.1016/j.jpain.2011.11.006 22277322

[87] Nijs J, Kosek E, Van Oosterwijck J, et al Dysfunctional endogenous analgesia during exercise in patients with chronic pain: to exercise or not to exercise? Pain Physician 2012;15:ES205–13. 22786458

[88] Littlewood C, Malliaras P, Chance-Larsen K Therapeutic exercise for rotator cuff tendinopathy: a systematic review of contextual factors and prescription parameters. Int J Rehabil Res 2015;38:95–106. 10.1097/MRR.0000000000000113 25715230

[89] Østerås B, Østerås H, Torstensen TA, et al Dose-response effects of medical exercise therapy in patients with patellofemoral pain syndrome: a randomised controlled clinical trial. Physiotherapy 2013;99:126–31. 10.1016/j.physio.2012.05.009 23219636

[90] Rathleff MS, Roos EM, Olesen JL, et al Exercise during school hours when added to patient education improves outcome for 2 years in adolescent patellofemoral pain: a cluster randomised trial. Br J Sports Med 2015;49:1–7. 10.1136/bjsports-2014-093929 25388552

[91] Cools AM, Michener LA Shoulder pain: can one label satisfy everyone and everything? Br J Sports Med 2017;51:416–7. 10.1136/bjsports-2016-096772 27806952

[92] Sloan TJ, Walsh DA Explanatory and diagnostic labels and perceived prognosis in chronic low back pain. Spine 2010;35:E1120–5. 10.1097/BRS.0b013e3181e089a9 20838269

[93] Smith BE, Thacker D, Crewesmith A, et al Special tests for assessing meniscal tears within the knee: a systematic review and meta-analysis. Evid Based Med 2015;20:88–97. 10.1136/ebmed-2014-110160 25724195

[94] May S, Littlewood C, Bishop A Reliability of procedures used in the physical examination of non-specific low back pain: a systematic review. Aust J Physiother 2006;52:91–102. 10.1016/S0004-9514(06)70044-7 16764546

[95] May S, Chance-Larsen K, Littlewood C, et al Reliability of physical examination tests used in the assessment of patients with shoulder problems: a systematic review. Physiotherapy 2010;96:179–90. 10.1016/j.physio.2009.12.002 20674649

[96] Seffinger MA, Najm WI, Mishra SI, et al Reliability of spinal palpation for diagnosis of back and neck pain: a systematic review of the literature. Spine 2004;29:E413–25. 15454722 10.1097/01.brs.0000141178.98157.8e

[97] Smith TO, Davies L, Donell ST The reliability and validity of assessing medio-lateral patellar position: a systematic review. Man Ther 2009;14:355–62. 10.1016/j.math.2008.08.001 18824392

[98] Smith TO, Hunt NJ, Donell ST The reliability and validity of the Q-angle: a systematic review. Knee Surg Sports Traumatol Arthrosc 2008;16:1068–79. 10.1007/s00167-008-0643-6 18841346

[99] Schulz KF, Grimes DA Blinding in randomised trials: hiding who got what. Lancet 2002;359:696–700. 10.1016/S0140-6736(02)07816-9 11879884

